# Machine learning for pattern and waveform recognitions in terahertz image data

**DOI:** 10.1038/s41598-020-80761-9

**Published:** 2021-01-13

**Authors:** Dmitry S. Bulgarevich, Miezel Talara, Masahiko Tani, Makoto Watanabe

**Affiliations:** 1 Research Center for Structural Materials, National Institute for Materials Science, 1-2-1 Sengen, Tsukuba, Ibaraki 305-0047 Japan; 2 Research Center for Development of Far-Infrared Region, University of Fukui, 3-9-1 Bunkyo, Fukui, 910-8507 Japan

**Keywords:** Imaging and sensing, Infrared spectroscopy, Scientific data

## Abstract

Several machine learning (ML) techniques were tested for the feasibility of performing automated pattern and waveform recognitions of terahertz time-domain spectroscopy datasets. Out of all the ML techniques under test, it was observed that random forest statistical algorithm works well with the THz datasets in both the frequency and time domains. With such ML algorithm, a classifier can be created with less than 1% out-of-bag error for segmentation of rusted and non-rusted sample regions of the image datasets in frequency domain. The degree of linear correlation between the rusted area percentage and the image spatial resolution with terahertz frequency can be used as an additional cross-validation criteria for the evaluation of classifier quality. However, for different rust staging measured datasets, a standardized procedure of image pre-processing is necessary to create/apply a single classifier and its usage is only limited to 1 ± 0.2 THz. Moreover, random forest is practically the best choice among the several popular ML techniques under test for waveform recognition of time-domain data in terms of classification accuracy and timing. Our results demonstrate the usefulness of random forest and several other machine learning algorithms for terahertz hyperspectral pattern recognition.

## Introduction

Terahertz/gigahertz time-domain spectroscopy (THz/GHz-TDS) is a promising nondestructive testing (NDT) technique for applications in various materials due to its harmless nature, longer radiation penetration depth for various materials, and the possibility of getting a rich structural and complex dielectric information in time and frequency domains^1,2^. THz/GHz-TDS imaging is inherently a hyperspectral technique with possibility of sliced imaging in frequency as well as in time domains since each image pixel contains a THz/GHz waveform^3,4^. In this respect, the typical spectral resolution of a classical THz-TDS system with mechanical delay stage is limited to ~ 0.8 GHz at ~ 0.5 THz due to the following reasons: (1) the duration of the temporal window brought by the laser repetition rate, (2) the length of the delay stage, and (3) the noise introduced by the fluctuations in THz radiation/detection^5^. In addition, time resolution is limited only by the fs-pulse duration and minimum mechanical delay stage step. Moreover, the spatial image resolution is governed by the Abbe diffraction limit with used THz wavelength and employed optics. In spite of the very attractive benefits and potentials of THz/GHz-TDS imaging, the associated large image data volumes and complex image contrasts could make the analysis and interpretation very difficult and time consuming.

In recent years, dramatic progress has been made in automated image pattern recognition techniques for automobile^6^, medical^7^, biological^8^, agricultural^9^, IT^10^, security^11^, and other applications. With suitable algorithm, image database, and powerful computers, the large volumes of image data can be classified in a short time with high accuracy^12,13^.

Apart from spectral recognition using various techniques in previous works^14–18^, statistical pattern recognition with Feed-Forward Artificial Neuronal Network (ANN), Convolutional Neural Networks (CNN), and Gaussian Mixture Model (GMM) were applied for sorting of recycling plastics by using THz frequency domain data^19^. Classification of breast cancer, if benign or malignant, from biomedical THz images with ANN and *K*-Nearest Neighbor (KNN) pattern recognition algorithms was also reported^20^. Just recently, KNN, Support Vector Machine (SVM), and Random Forest (RF) were compared in terms of total classification accuracy and recognition ability for the diagnosis of traumatic brain injury and results showed that RF was the best algorithm among the three algorithms under study^21^. In a previous study^22^, SVM was observed to be effective in distinguishing patterns associated with ribonucleic acid and various powdered substances hidden inside noisy biomedical measurements. Minimum Distance classifier and ANN methods were implemented for identification of explosives and bio-chemical materials from THz images at different frequencies^23^. Moreover, a deep learning algorithm was used for classification of THz images^24,25^. In addition, a simple neural network for resolving of coating thickness^26^ and a back-propagation (BP) neural network and SVM for conducting regression analysis for the characterization of thermal barrier coatings with THz-TDS were realized^27^. Besides ML algorithms^28^, various segmentation methods^29,30^ were applied for automated detection of concealed objects from THz images.

However, there is still very limited literature on the application of ML techniques in nondestructive testing (NDT) using pattern or waveform recognition in THz-TDS datasets. Just recently, a classical BP neural network algorithm and a novel extreme learning machine (ELM) algorithm were employed for NDT of measuring film thickness from THz time domain data^31^. In this respect, we demonstrate the feasibility of pattern recognition in THz-TDS images of rusted steel with RF machine learning algorithm^32,33^. Such analysis was conducted in frequency domain which is more widely used in NDT applications due to higher achievable spatial resolution compared to time domain imaging. Time domain imaging is typically employed for NDT measurements of sample thickness. Hence, the possibility of THz waveform recognition is also outlined with various ML methods. Here, it should be noted that applications of such techniques are novel for THz-TDS imaging, NDT, and materials science fields.

## Experimental

For pattern recognition, sample preparations were carried out by wetting the steel plate, with cross-cuts in paint layer, with 3% NaCl solution and sealing it in container with 100% humidity at 23 °C to rust. The prepared samples were periodically taken out and dried naturally. THz-TDS imaging was performed by using a broadband Pulse IRS-2000 (AISPEC) THz-TDS system. The linearly polarized THz beam was focused on the sample surface using an optical reflection stage (≈ ± 10.3° maximum cone angle). The THz beam profile with our THz-TDS system was previously reported^34^. The samples were then raster scanned with 0.2 × 0.2 mm^2^ spatial pixel resolution using a mapping mechanical stage in the focal plane of such reflection stage. The waveforms were collected for spectral analysis with 4096 data points at ~ 4.2 fs time intervals, i.e. with ~ 58 GHz spectral image resolution. Note that we studied the non-hidden rust patterns with THz-TDS in order to compare the image segmentation results with that of visible light optical images. For waveform recognition experiments, we used the aperture array of Siemens-star apertures microfabricated in thin-film Al on a thick Si substrate, which was previously described in literature^35^.

The open source FIJI software package with Trainable Weka Segmentation plugin was applied for image dataset pre-processing, analysis, and segmentation on two-CPU 6128 Opteron Workstation with 128 GB RAM^36–40^. ML on training/test datasets with THz waveforms was performed by using the open source WEKA software package with collection of ML algorithms^41^.

## Results and discussion

### Machine learning in frequency domain

**Figure 1:**
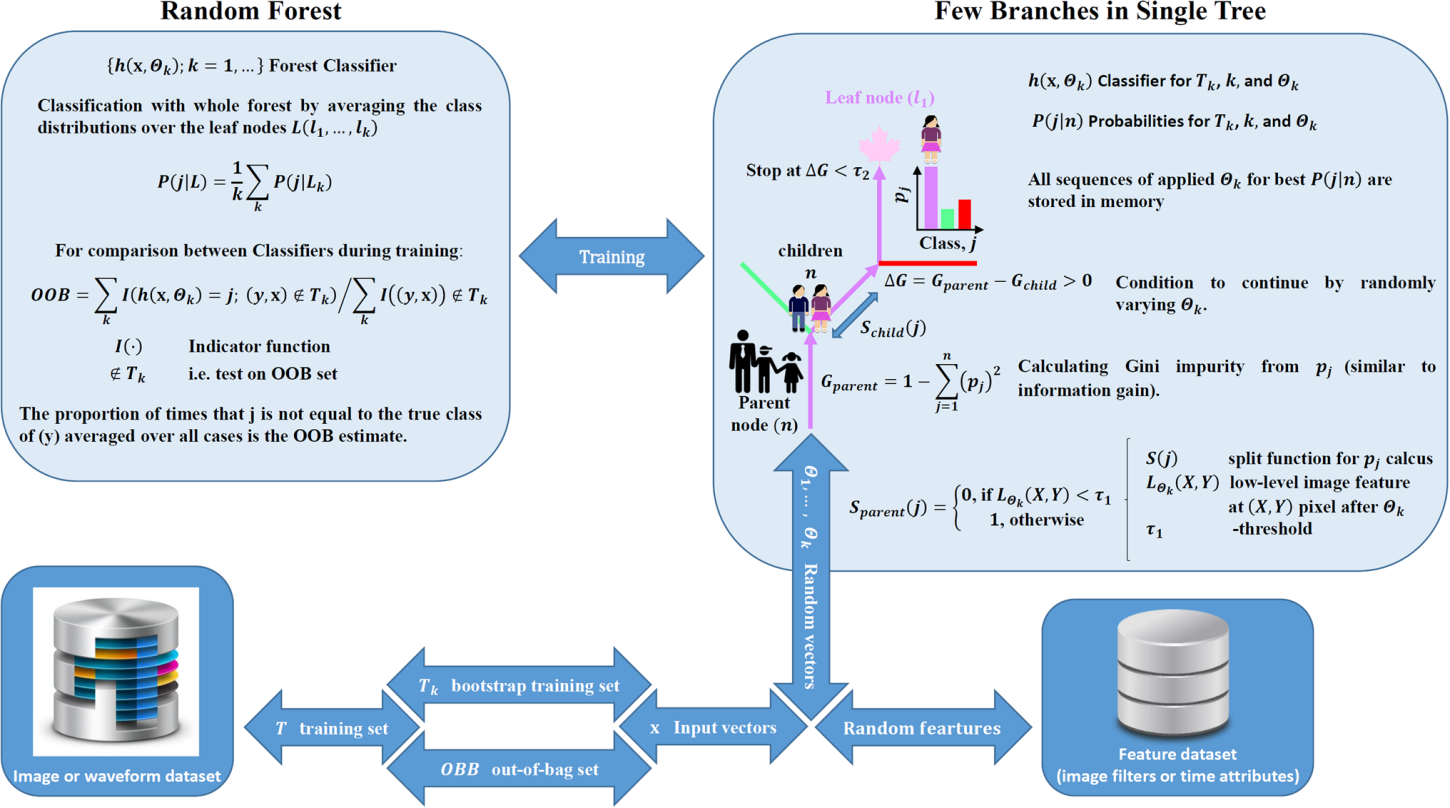
Schematic diagram of the principle behind RF classifier for pattern and waveform recognitions.

Figure 1 shows the general outline of the RF algorithm for image segmentation on several classes (${\text{j}}$), i.e. on rusted and non-rusted regions in our images. The basic idea is to generate the forest from individual decision tree ($k$) classifiers [$h({\mathbf{x}},\;\Theta_{k} )$], which will store the information on appropriate sequences of image filter applications, optimized threshold parameters for split function [$S(j)$] and leaf node highest probabilities ($P$) used to assign each image pixel to particular ${\text{j}}$. Here, ${\Theta }_{k}$ are the feature vectors obtained by transforming the image input vectors (${\mathbf{x}}$) at each $k$ node with image filters/kernels. The collection of such [$h({\mathbf{x}},\;\Theta_{k} )$] from all $k$ is the RF classifier [$\left\{ {h({\mathbf{x}},\;\Theta_{k} );\;k = 1, \ldots } \right\}$].

In this ensemble-type supervised learning, the user has to choose the image or images from the dataset for training process in order to create the RF classifier: the program for automatic segmentation/classification of other images with the same ${\text{j}}$ present. This step requires several user interactions: providing the training data set (${\text{T}}$) with good examples of image areas/pixels for every ${\text{j}}$, forming the image filter dataset, and defining the forest scale such as number of trees, their maximum depth or calculation precision. In RF algorithm, the bootstrap data ($T_{k}$) for each decision tree (${\text{k}}$) construction are chosen from ${\text{T}}$ at random, but with overlays. During this process, ~ 1/3 of ${\text{T}}$ are left unused to form the out-of-bag (OOB) dataset for later estimation of RF classifier error. Note that levelling-off the classification accuracy for forests with more than ~ 100–200 trees^13,42,43^ is well-documented.

At the classification stage on training image/images, the pixels that arrives to $L_{k}$ after all filtering and binary tests stages are assigned to $P(j{|}L_{k} )$ in each $k$, which were obtained at $k$ construction stage. To make a decision on ${\text{j}}$ assignment for each pixel, every $k$ casts a unit vote for the most popular class. These votes from all $k$ cast a unit vote too. As a result, each pixel is finally classified to the particular ${\text{j}}$. Then, the user can optimize the RF classifier by changing/adding the training areas, applied filters, and forest parameters to minimize the OOB error and to improve the visual segmentation quality. Finally, the RF classifier can be saved and used on other images. The ensemble effect and de-correlation of trees due to random bootstrapping and filtering make the RF classifiers unbiased and very stable to image noise compared to other statistical methods. However, they may also have some limitations, which will be discussed below.

**Figure 2:**
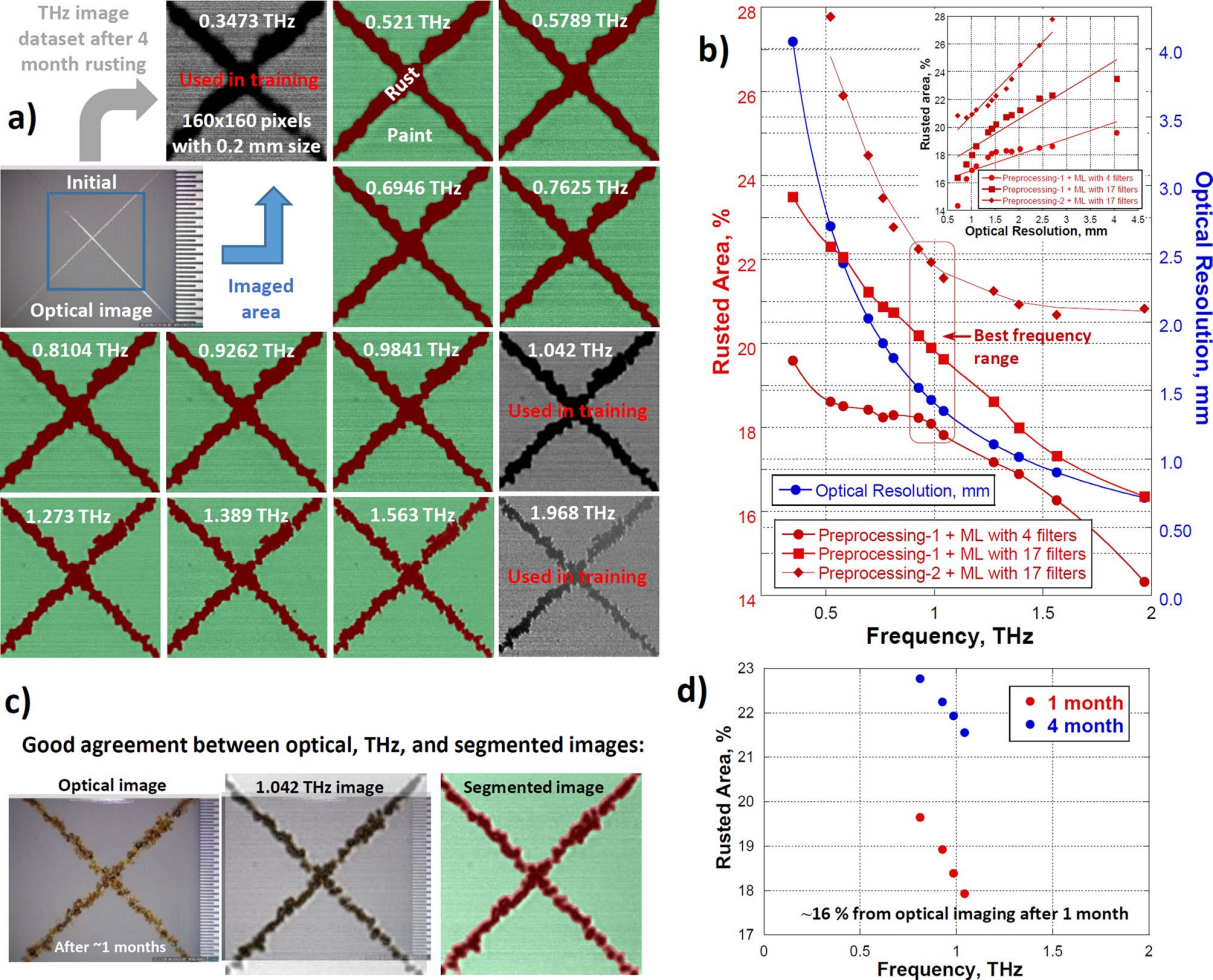
Analysis with ML in frequency domain. (**a**) Overlays of reflection THz and auto-segmented images of painted, cross-scratched, and subsequently rusted steel sample showing colored rusted and painted regions. The gray pictures are optical images of non-rusted sample and reflection THz images used for ML classifier training. The indicated frequencies correspond to a particular image in the spectral dataset. (**b**) Frequency vs % Rusted Area comparison of the different ML pre-processing conditions showing the classifier quality depends strongly on user-supervised training for lower OOB error, better overlays and correlation with optical resolution. (**c**) Sample optical, THz and segmented images showing good agreement in classifying rusted and painted regions. (**d**) Graphical representation of the good agreement between the optical resolution and the classifier quality.

Figure 2a shows the optical and THz images of the initial and rusted samples, respectively. To create the RF classifier with OOB error below 1% for particular rusting time, it is necessary to train it on THz images with different spatial resolutions (THz frequencies) due to different noise and contrast levels within the image spectral dataset. Then, ${\text{T}}$ with examples of two classes (rust and paint) is selected from images at ~ 0.3, ~ 1, and ~ 2 THz. Figure 2b demonstrates the difference between several RF classifiers built with different pre-processing protocols to address such noise and contrast variations as well as with different number of used image filters for particular RF buildup. In the case of classifier with 4 filters, the following ones were employed: Gaussian Blur, Hessian, Sobel, and Difference of Gaussians. For the case with 17 filters, Membrane Projections, Mean, Maximum, Anisotropic Diffusion, Lipschits, Gabor, Entropy, Variance, Minimum, Median, Bilateral, Kuwahara, and Neighbors filters were added.

As shown in Fig. 2b, not only the absolute values of rusted area percentage at fixed frequencies were different between classifiers, but the shape of frequency dependence also varies. The minimum variations between classifiers were observed at ⁓ 1 THz since spatial resolution or signal-to-noise ratio decreased at lower or higher frequencies, respectively. In principle, due to the nature of RF, the use of larger number of different filters/kernels for ${\Theta }_{k}$ should lead to better quality classifier. However, by using OOB errors and visual inspection alone, it is difficult to judge whether the improvements of classifier quality depend on the preprocessing protocols or the number/type of image filters being used since segmentations seemed to be good anyway.

In this respect, the correspondence between frequency dependences of rusted area percentage and optical resolution could serve as an additional cross-validation criterion for classifier quality (see comparison in Fig. 2b and in insert). Image pre-processing protocols which include (1) background brightness correction, (2) background correction with sliding paraboloid of 150 pixel radius, (3) noise reduction by image smoothing with Gaussian blur filter (σ = 1), and (4) contrast normalization with analysis of the image histograms as well as the training of classifiers with 17 image filters^39^ have resulted to reasonable classifier quality (see plots with Preprocessing-2 in Fig. 2b).

The performance of the resulted RF classifier is compared in Fig. 2c,d with the classifier built on the corresponding optical image. The difference in the obtained rusted area percentage estimated from both the optical and THz images was reasonable due to the higher optical resolution with visible light. Currently, the most accurate results were obtained at ⁓ 1 THz (see Fig. 2b). Note that the same RF classifier (with Preprocessing-2) created on a 4-month rusting dataset was applied on the THz image datasets for 1-month rusting time. This demonstrates the feasibility of creating a single classifier for analyses of different rust staging THz image datasets, which is practically useful for NDT.

### Machine learning in time domain

**Figure 3:**
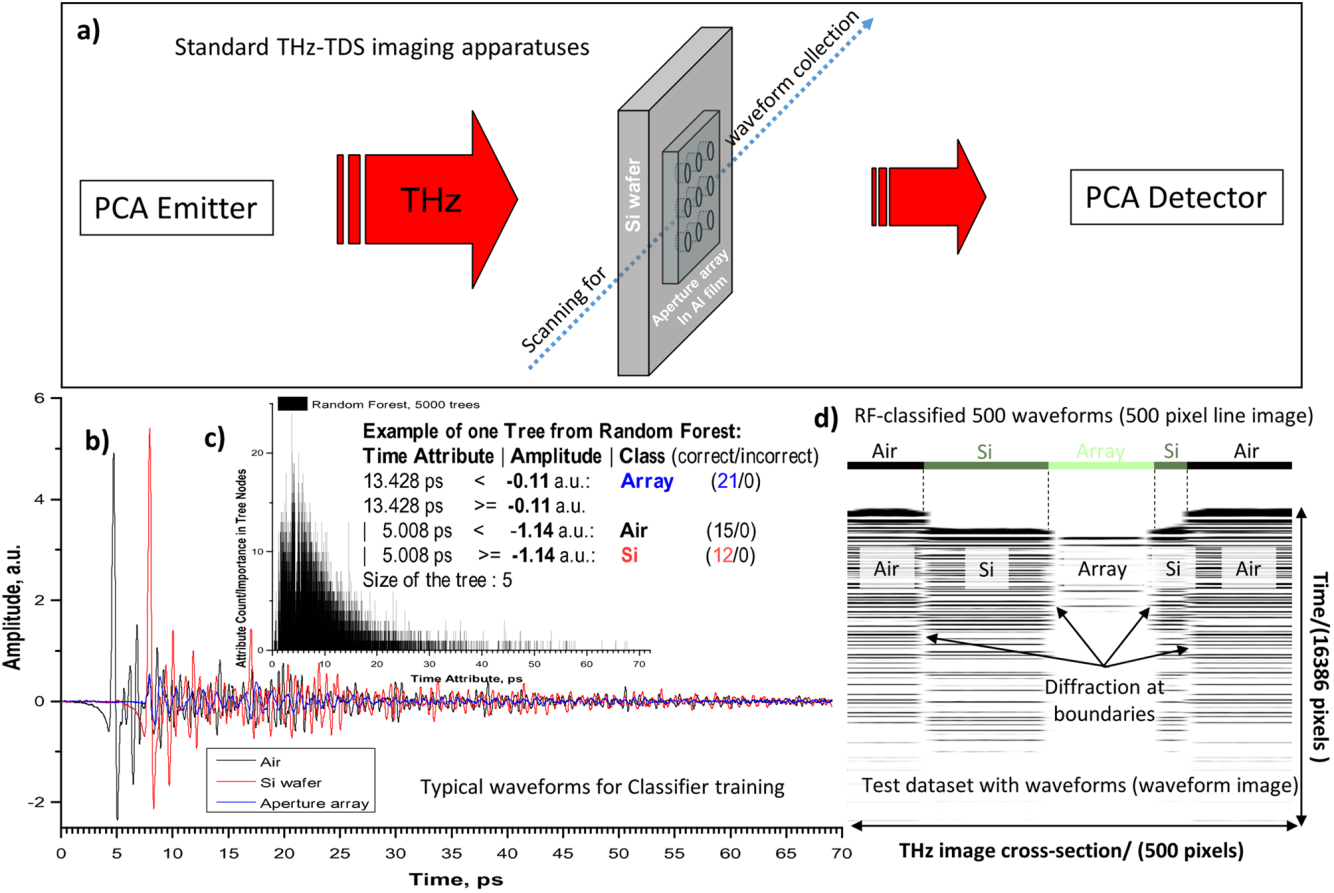
Analysis with ML in time domain. (**a**) Schematic diagram for waveform collection showing the Al aperture array filter on Si substrate configuration in the THz-TDS imaging system. (**b**) Examples of transmission waveforms taken from THz-TDS image of the Al aperture array filter on Si substrate. For classifier training, 16 waveforms for each class (Air, Si, and Array) were used. (**c**) Time attribute vs Attribute count graph showing attribute importance evaluation with RF on train dataset. (**d**) Comparison of class assignments by RF classifier (top line image) and waveform amplitude image of the test dataset (lower grey image). The latter one was obtained by converting all 500 waveforms across the THz-TDS image (line of 1 pixel height) to the text image matrix with waveform amplitude value vs time in each column (waveform image). The dotted vertical lines are RF-estimated class boundaries.

Figure 3a shows the basic experimental setup used for THz-TDS imaging of Al aperture array described elsewhere^35^. For ML, 500 waveforms across a 12 × 12 mm array on Si wafer (30 × 30 mm) in air were used. Each waveform with 16,384 data points corresponds to a single pixel with 0.1 × 0.1 mm spatial size from image line of 500 pixels. These waveforms belong to three classes: Air, Si wafer, and Aperture array. Such sample was used for our proof of concept since these classes have distinct waveforms as shown in Fig. 3b. For classifier training, 16 waveforms of each class were selected, which formed the training dataset.

Table 1 compares the performance of several ML techniques on such training dataset with tenfold stratified cross-validation, i.e. it invokes the learning algorithm 11 times, once for each fold and then a final time on the entire training dataset. The classifier settings were based on known behaviors of these algorithms. Note that diagonal and off-diagonal elements in the provided confusion matrixes correspond to the number of correct and incorrect classifications, respectively. Among the tested classifiers, RF demonstrated not only 100% correct classification of training dataset, but also exhibited the best overall training/testing time of the entire dataset with 500 waveforms.

**Table 1:** List of tested ML classifiers for THz waveform recognition with provided training/testing performances.

Classifier name	Classifier main used/optimized parameters	Tenfold validation on train dataset	Time (s)	
		Confusion matrix, correct classification	Training	Testing
Random Forest (RF)	100 trees, int(log_2_(features) + 1) randomly chosen attributes	$$\begin{array}{*{20}c} {Air} & {\quad Si} & {\quad Array} & {\quad {\mathinner{\mkern2mu\raise1pt\hbox{.}\mkern2mu \raise4pt\hbox{.}\mkern2mu\raise7pt\hbox{.}\mkern1mu}} } \\ {16} & {\quad 0} & {\quad 0} & {\quad Air} \\ 0 & {\quad 16} & {\quad 0} & {\quad Si} \\ 0 & {\quad 0} & {\quad 16} & {\quad Array} \\ \end{array}$$	1.20	5
Logistic (Log)	10^–8^ ridge value, full feature-class weights optimization		627	6
Support vector machine (SVM)	Log calibrator (see above), the linear polynomial kernel		0.45	7
Naive Bayes (NB)	None		0.27	36
k-Nearest neighbors (KNN)	1 neighbor, linear NN Euclidean distance search		0.02	22
Single-layer perceptron (SP)	1 hidden layer, 6 nodes, 0.3 learning rate, 0.2 momentum	100%	289	11
k-Means clustering (k-M)	5 clusters, Euclidean distance function	$$\begin{array}{*{20}c} {Air} & {\quad Si} & {\quad Array} & {\quad {\mathinner{\mkern2mu\raise1pt\hbox{.}\mkern2mu \raise4pt\hbox{.}\mkern2mu\raise7pt\hbox{.}\mkern1mu}} } \\ {14} & {\quad 0} & {\quad 0} & {\quad Air} \\ 0 & {\quad 15} & {\quad 0} & {\quad Si} \\ 0 & {\quad 0} & {\quad 16} & {\quad Array} \\ \end{array}$$	0.6	7
		~ 94% (with k = 5)		

Figure 3c shows the plot of attribute counts from 5000 RF trees, which reveals the internal attribute selection in the process of basic RF classifier build-up. The attributes between ⁓ 2–15 ps have higher counts in RF trees since in this delay time range, largest amplitude differences between waveform classes are present. The insert in Fig. 3c provides example of one logical tree from RF with used time attributes, selected thresholds, and assigned classes.

Figure 3d demonstrates the correspondence between RF estimated and experimental class boundaries. The bold colored line is RF results. Below this line, the waveform image is shown for comparison. Each pixel intensity in vertical column is the amplitude value at particular delay time for a single waveform. The 500 columns correspond to the number of waveforms in the test dataset. As it can be seen, RF placed the class boundaries quite accurately at diffraction boundaries in the experimental THz image.

**Figure 4:**
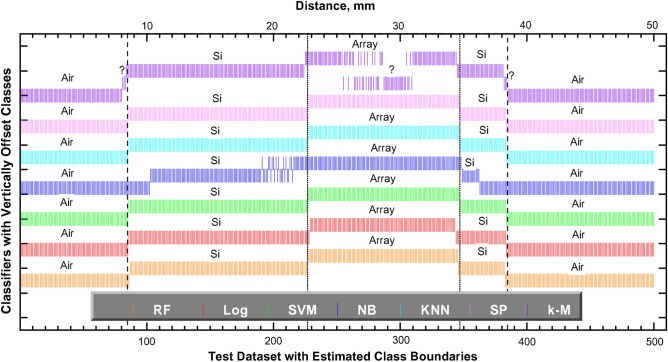
Performance comparison of the different ML classifiers on the test dataset (see also Table 1) by comparing class boundaries of these classifiers with the actual Array and Si dimensions, which are shown by dotted and dashed black lines, respectively. The question marks indicate waveforms which cannot be assigned to any of three classes (Si, Array, or Air) with ML classifier.

To compare the accuracy of estimated class boundaries between different ML techniques, Fig. 4 plots class assignment for each waveform from the test dataset. In Fig. 4, each vertical color bar corresponds to the single waveform from each pixel in the image line, which is then assigned to a particular class with well-trained ML classifier. The vertical offsets were added for clarity. The *k*-M classifier exhibited the worst classification on train/test datasets for $k = {\text{j}}$ while the best classification was obtained for $k = 5$, i.e. for $k > {\text{j}}$ (see Table 1). However, this classifier is still behind other tested ML methods in terms of classification performance. Moreover, NB, which is another ML algorithm being tested in this study, also demonstrated poor accuracy in spite of good training results. This is actually expected due to the nature of NB classifier, that is, the assumption of independent and equal contribution to the outcome of each feature/time.

In principle, multi-parameter optimizations could still be performed for *k*-M classifier using MultiSearch meta-method in WEKA by optimizing the arbitrary number of user defined parameters and their ranges. However, we still need to use attribute selection/filtering with other tools. We did not proceed rigorously with *k*-M classifier tuning due to cumbersome efforts compared to already achievable 100% training accuracy with only simple settings established for RF and other well performing algorithms (see Table 1 and Fig. 4). Moreover, the state-of-the-art Auto-WEKA can be used for tuning of 789 hyperparameters from classification algorithms built into the WEKA software package^44,45^. Depending on the requested accuracy, given time, and provided PC resources, the output is the best classifier or classifier list with information on found hyperparameters. These tools could be useful and they are available for other more challenging training datasets.

## Conclusions

In principle, the results of this study could be used as a basis for more complex classification/analysis. It can be used for the automatic classification and analysis of painted/covered patterns and partial waveforms (wavelets). This may found its applications in industrial/security on-the-fly NDT together with THz/GHz and THz/GHz-TDS imaging/analysis. RF outperformed k-N and NB in overall accuracy on waveform training/testing datasets. It also outperformed all classifiers in overall training/testing time. Above all, RF typically does not require any parameter tuning to get good results if to use with 100–200 or more trees in a forest, making it as a user-friendly and a robust method while mostly outperforming other ML techniques on same training/testing datasets^46,47^. Apart from that, Log, SVM, KNN, and SP (MP) are also very capable classifiers for waveform recognition task. Moreover, RF also produced very good results for image segmentation. However, detailed comparison with other ML methods, especially with convolutional neural networks (CNN), is still needed in the future.
